# Urinary Biomarkers for Radiation Cystitis: Current Insights and Future Directions

**DOI:** 10.3390/ijms27020565

**Published:** 2026-01-06

**Authors:** Rani Mahyoob, Bernadette M. M. Zwaans

**Affiliations:** 1 Department of Urology, Corewell Health William Beaumont University Hospital, Royal Oak, MI 48073, USA; rani.mahyoob@corewellhealth.org; 2 Department of Urology, William Beaumont School of Medicine, Oakland University, Rochester, MI 48073, USA

**Keywords:** radiation cystitis, bladder, urinary biomarkers

## Abstract

Radiation cystitis (RC) is a clinically challenging and often progressive complication of pelvic radiotherapy, marked by urothelial injury, vascular dysfunction, chronic inflammation, and fibrotic remodeling. Early diagnosis remains elusive due to nonspecific symptoms and the absence of validated molecular tools. As a biofluid in direct contact with the irradiated bladder, urine offers a unique molecular window into RC pathogenesis. In this review, we synthesize the current landscape of urinary biomarkers associated with the acute, latent, and chronic phases of RC, including inflammatory cytokines, oxidative stress products, epithelial injury markers, extracellular vesicles, microRNAs, proteomic signatures, and metabolomic alterations. We also integrate emerging mechanistic insights such as DNA damage responses, ROS generation, mitochondrial dysfunction, urothelial barrier disruption, senescence-associated secretory phenotypes, hypoxia-driven vascular injury, and profibrotic TGF-β signaling, all of which contribute to the release of urinary analytes. By linking phase-specific molecular pathways with corresponding urinary signatures, we highlight opportunities to leverage urine-based measurements for early detection, risk stratification, severity assessment, and therapeutic monitoring. A deeper understanding of the molecular mechanisms shaping urinary biomarker profiles will be essential for advancing precision diagnostics and improving long-term outcomes for patients with radiation cystitis.

## 1. Introduction

Radiation cystitis (RC) is a clinically significant and often debilitating complication of pelvic radiotherapy. It is most frequently observed in prostate cancer survivors, but also occurs in patients treated for cervical, rectal, and bladder cancers [1]. The incidence of RC varies widely, ranging from 23% to 80%, depending on treatment dose, fractionation schedules, concurrent therapies, and follow-up duration [2]. Severe forms, such as hemorrhagic cystitis, are less common but occur in up to 5–8% of patients, which sometimes takes many years after treatment completion to manifest [3]. Clinically, RC symptoms can include hematuria, dysuria, urinary urgency, urinary incontinence and frequency. In advanced cases, severe bladder fibrosis, reduced bladder capacity and hematuria can result in chronic morbidity, recurrent hospitalizations, and may ultimately require cystectomy [4]. These outcomes not only impair quality of life and can be life threatening but also impose a substantial burden to the health care system.

The pathogenesis of RC is complex, involving a triphasic process with distinct acute, latent and chronic components [5,6]. The acute phase is dominated by urothelial injury, inflammation, and vascular congestion, leading to irritative lower urinary tract symptoms during or shortly after radiotherapy [7]. In contrast, the chronic phase is characterized by progressive fibrosis, vascular rarefaction, ischemia, and impaired urothelial regeneration, changes that may manifest clinically months to decades later [8]. The latent phase, during which patients tend to be symptom-free, is less well understood. Importantly, these biological processes generate measurable molecular signatures including cytokines, chemokines, oxidative stress mediators, and vascular injury proteins that could serve as biomarkers for disease detection and monitoring.

Diagnosis of RC remains challenging because its symptoms overlap with those of other bladder pathologies, such as urinary tract infections, bladder malignancy, or interstitial cystitis [4]. Currently, diagnosis is often based on clinical exclusion, supported by cystoscopy and imaging when necessary [9]. Therapeutic options are limited and largely supportive, ranging from bladder irrigation and intravesical instillations to hyperbaric oxygen therapy and surgical interventions in refractory cases [10]. Thus, there is an urgent need for tools that can facilitate early detection, stratify risk, and monitor disease progression effectively.

In this context, urine represents an attractive biofluid for biomarker discovery and clinical translation. It can be collected non-invasively, repeatedly, and at low cost, making it feasible for longitudinal monitoring in large cohorts. Unlike blood, which reflects systemic alterations, urine is in direct contact with the bladder urothelium for extend amounts of time, offering a unique opportunity to capture local pathophysiological changes. This “liquid biopsy” of the bladder may reveal molecular correlates of urothelial damage, vascular compromise, oxidative stress, and fibrosis [11]. Furthermore, urine is already widely used in clinical practice for diagnostic testing (e.g., infections, hematuria evaluation), which could facilitate adoption of biomarker-based assays once validated.

Emerging studies lend support to the feasibility of urinary biomarkers in RC. Zwaans et al. reported that prostate cancer survivors with symptomatic RC showed elevated urinary levels of fibrotic and vascular proteins such as tissue inhibitors of metalloproteinases (TIMP-1, TIMP-2), plasminogen activator inhibitor-1 (PAI-1), and vascular endothelial growth factor A (VEGF-A), highlighting the contribution of fibrosis and vascular injury to disease pathogenesis [12]. Similarly, the RABBIO study identified urinary cytokines, including macrophage colony-stimulating factor (M-CSF) and macrophage inflammatory protein-1 (MIP-1), as potential indicators of acute bladder toxicity during radiotherapy [13]. More recently, metabolomic profiling studies have identified candidate small-molecule signatures such as betaine, tartrate, and homocarnosine associated with early RC symptoms [14]. Collectively, these findings illustrate the promise of urinary biomarkers to capture different biological aspects of RC, spanning inflammation, oxidative stress, vascular injury, and fibrosis.

Despite encouraging progress, the field remains at an early stage. Most studies to date have been exploratory, involving relatively small and heterogeneous patient cohorts. Methodological differences in urine collection, storage, and analysis contribute to variability in reported findings [15,16,17,18,19,20,21]. Moreover, there is limited validation of biomarker candidates across independent cohorts, and little is known about their predictive value for long-term outcomes [22,23,24,25]. A further challenge lies in distinguishing RC from other bladder pathologies that present with overlapping clinical features and may similarly alter urinary molecular profiles [26,27,28]. As a result, no urinary biomarker has yet entered routine clinical practice for RC management [12,29].

This review provides a comprehensive synthesis of current insights into urinary biomarkers for RC. We begin with an overview of the biological mechanisms of RC, followed by a structured summary of urinary biomarker candidates and their supporting evidence. Lessons from biomarker research in other radiation-affected organs are considered, alongside emerging technologies such as omics profiling and machine learning. Finally, we highlight key challenges to clinical translation and propose strategies for advancing urinary biomarkers toward standardized use in precision medicine approaches for RC. This article is intended as a narrative, mechanistic review synthesizing preclinical, translational, and emerging clinical evidence on urinary biomarkers in radiation cystitis, rather than a formal systematic review with predefined inclusion or exclusion criteria.

## 2. Pathophysiology of Radiation Cystitis

Radiation cystitis (RC) develops through a complex, time-dependent sequence of tissue injury and attempted repair, commonly categorized into acute, latent, and chronic phases [30]. Although these phases are described separately, they represent a continuous biological cascade initiated by ionizing radiation exposure. Each stage reflects distinct but overlapping mechanisms involving urothelial damage, vascular dysfunction, immune activation, oxidative stress, and stromal remodeling, all of which contribute to the urinary biomarker profiles observed in RC [31].

### 2.1. Acute Phase

**Figure 1:**
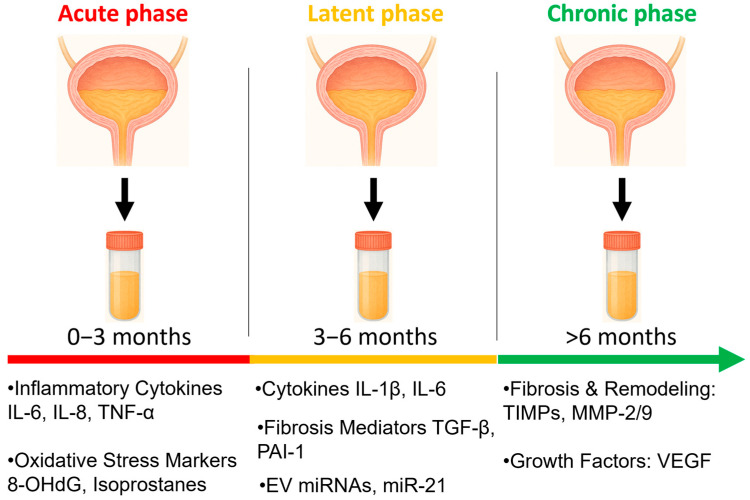
Pathophysiological Timeline of Radiation Cystitis and Biomarker Emergence. Icons were generated using Microsoft Copilot Smart (GPT-5, 2025).

The acute phase usually manifests during or within the first weeks after radiotherapy. It is driven primarily by direct radiation-induced DNA damage in urothelial cells, including double-strand breaks that activate ATM/ATR signaling and p53-mediated apoptosis [32,33]. This result in loss of the protective glycosaminoglycan (GAG) layer, epithelial denudation, and increased urothelial permeability. These changes allow toxic urinary solutes to penetrate deeper layers of the bladder wall, amplifying tissue injury. These epithelial injuries trigger the release of danger-associated molecular patterns (DAMPs) such as HMGB1, ATP, and heat-shock proteins, which activate Toll-like receptors (TLR2/TLR4) and RAGE on resident immune and stromal cells [34,35]. Activation of these receptors drives NF-κB–mediated production of IL-1β, IL-6, and TNF-α (Figure 1), amplifying the inflammatory response. This is accompanied by early infiltration of neutrophils and macrophages, and robust generation of reactive oxygen species (ROS) from both mitochondrial dysfunction and NADPH oxidase activation [36]. The acute inflammatory response is characterized by infiltration of neutrophils and macrophages, upregulation of pro-inflammatory cytokines (e.g., IL-1β, IL-6, TNF-α) (Figure 1), and release of reactive oxygen species (ROS), which collectively contribute to mucosal edema, hyperemia, and irritative urinary symptoms [9,37,38].

**Figure 2:**
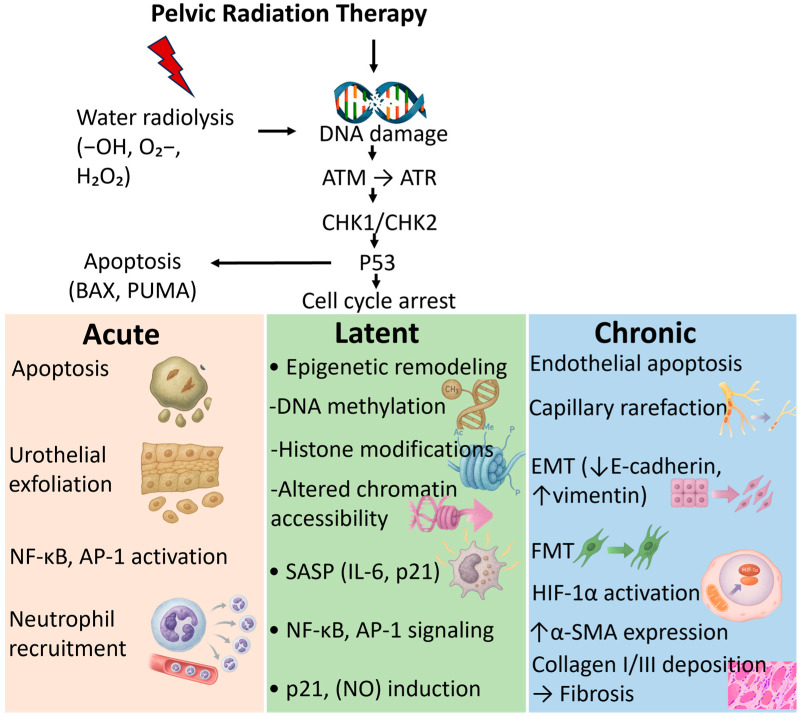
Molecular mechanisms underlying the development of radiation cystitis. Icons were generated using Microsoft Copilot Smart (GPT-5, 2025).

Although acute RC is often self-limiting, these early molecular events set the stage for long-term damage. Persistent inflammation, oxidative stress and impaired barrier repair may lead to chronic remodeling processes if repair mechanisms fail (Figure 2) [39].

### 2.2. Latent Phase

The acute phase is followed by a symptom-free latent phase that can span months to decades following pelvic radiotherapy, during which subclinical tissue damage silently progresses before overt symptoms emerge. Despite clinical quiescence, molecular and structural injury continues to progress during this silent interval. A key event in this phase is radiation-induced microvascular injury, mediated in part by acid sphingomyelinase–ceramide signaling, which promotes endothelial apoptosis and capillary rarefaction [40,41]. This phase is marked by insidious microvascular injury, urothelial stem cell depletion, and low-grade inflammation that fail to resolve, ultimately tipping the balance toward chronic fibrosis and ischemia. Histologically, obliterative endarteritis and progressive extracellular matrix deposition are hallmarks of this delayed pathology, often culminating in bladder wall thickening, reduced compliance, and hemorrhagic episodes [41]. Importantly, patients may remain asymptomatic during this interval, making early detection challenging. Biomarkers such as elevated urinary TIMP-1, PAI-1, and VEGF-A, reflecting tissue and vascular remodeling, have been associated with this latent remodeling phase. This suggests that molecular surveillance could identify patients at risk before clinical deterioration. Understanding the biology of this silent progression is critical for developing preventive strategies and biomarker-guided interventions that intercept RC before irreversible damage occurs [12].

Understanding the biology of this silent phase is crucial for developing preventive or early intervention strategies targeting latent-stage remodeling [42].

### 2.3. Chronic Phase

The chronic phase of RC may occur anywhere from several months to decades after completing radiotherapy, sometimes following a latency period without symptoms. It is characterized by progressive fibrosis, vascular compromise, and impaired tissue regeneration. Radiation-induced injury to the microvasculature, often termed obliterative endarteritis, reduces blood flow, leading to tissue hypoxia and ischemia [12]. Hypoxia, in turn, activates fibrogenic signaling pathways, most notably transforming growth factor-beta (TGF-β), connective tissue growth factor (CTGF), and vascular endothelial growth factor (VEGF), which drive fibroblast activation and extracellular matrix (ECM) deposition [8,42]. These processes culminate in bladder wall thickening, reduced compliance, and, in severe cases, hemorrhagic cystitis due to fragile neovascularization. In addition, Wnt/β-catenin signaling and the activity of collagen-crosslinking enzymes such as lysyl oxidase (LOX) and LOXL2 contribute to irreversible bladder wall stiffening and structural distortion [43]. TGF-β–driven fibroblast activation leads to increased urinary detection of ECM-related biomarkers such as collagen fragments, MMP-9, and LOX activity [44].

At the cellular level, chronic RC is marked by apoptosis and senescence of urothelial cells, loss of stem cell regenerative capacity, and sustained activation of myofibroblasts [45]. The resulting imbalance between injury and repair can lead to recurrent hematuria, fibrosis, and bladder dysfunction. Importantly, these pathophysiological processes generate detectable urinary molecular signatures, including oxidative stress markers (e.g., 8-OHdG), vascular injury proteins (e.g., VEGF, vWF), and fibrotic mediators (e.g., TIMPs, PAI-1) [12,14]. Senescent urothelial and stromal cells release SASP factors (IL-6, IL-8, GM-CSF, MCP-1), which enter urine and represent a major source of inflammatory biomarkers in both the latent and chronic phases (Figure 2) [46]. Collectively, these biomarkers reflect the interplay between chronic ischemia, fibroblast activation, and dysregulated tissue repair.

This schematic shows how RC progresses from acute to chronic phases, with corresponding urinary biomarkers mapped to each stage.

Pelvic radiation initiates water radiolysis, generating reactive oxygen species (–OH, O_2_–, H_2_O_2_) and inducing DNA damage or directly strikes DNA in urothelial and stromal cells. Activation of the DNA damage response (ATM → ATR → CHK1/CHK2) and p53 signaling leads to apoptosis and cell-cycle arrest. These upstream events give rise to phase-specific molecular pathologies. Acute phase: urothelial apoptosis, epithelial exfoliation, activation of NF-κB and AP-1 transcriptional pathways, and neutrophil recruitment. Latent phase: epigenetic remodeling, including DNA methylation, histone modifications, and altered chromatin accessibility, alongside SASP mediator production (e.g., IL-6, p21), persistent NF-κB/AP-1 signaling, and p21/nitric oxide (NO) induction. Chronic phase: endothelial apoptosis, capillary rarefaction, epithelial–mesenchymal transition (EMT; ↓E-cadherin, ↑vimentin), fibroblastmyofibroblast transition (FMT), HIF-1α activation, α-SMA upregulation, and collagen I/III deposition culminating in bladder wall fibrosis. Together, these mechanisms illustrate the progressive molecular cascade that drives the transition from early epithelial injury to chronic fibrotic remodeling in radiation cystitis.

### 2.4. Relevance to Biomarker Discovery

Understanding the sequential biology of RC is crucial for biomarker development. Biomarkers associated with the acute phase (e.g., cytokines, chemokines, oxidative stress mediators) may enable early detection, assessment of someone’s risk profile for developing RC, or monitoring of acute tissue toxicity, whereas chronic-phase biomarkers (e.g., fibrotic and angiogenic proteins) may help predict long-term outcomes and guide therapeutic interventions. Importantly, distinct biomarker signatures for acute versus chronic RC could facilitate disease staging, prognosis, and personalization of treatment [13].

## 3. Types of Urinary Biomarkers Studied

Unless otherwise specified, biomarker associations discussed below primarily derive from preclinical models, exploratory human cohorts, or associative clinical studies, and should not be interpreted as evidence of direct causality or validated clinical utility. The urinary compartment is uniquely suited for biomarker discovery in RC. Because urine directly interfaces with the irradiated urothelium, it captures molecular signals associated with epithelial injury, endothelial dysfunction, oxidative stress, immune activation, and stromal remodeling [47]. Urinary analytes, such as soluble proteins, cytokines, lipids, metabolites, nucleic acids, and extracellular vesicles (EVs), thus reflect both acute epithelial barrier disruption and progressive late-phase fibrosis. Importantly, many of these analytes originate from specific molecular pathways activated by ionizing radiation, including DNA damage responses, DAMP-mediated inflammation, hypoxia-induced angiogenic signaling, and TGF-β–associated fibrogenesis [13]. These mechanistic underpinnings provide the rationale for multiple urinary biomarker classes in RC.

### 3.1. Inflammatory Biomarkers

Radiation triggers sterile inflammation of the bladder mucosa through DAMP release (e.g., HMGB1, ATP, heat-shock proteins) and activation of TLR2/TLR4 and RAGE on urothelial and immune cells [48]. These receptors converge on NF-κB, promoting transcription of IL-6, IL-1β, TNF-α, CXCL8/IL-8, and MCP-1/CCL2. Many of these cytokines are readily detectable in urine and represent proximal readouts of inflammatory activation [12] (Table 1). Studies have measured urinary concentrations of interleukins (notably IL-6 and IL-8), TNF-α, MCP-1/CCL2, and other chemokines [49,50,51,52,53,54,55]. Elevated urinary IL-6/IL-8 have been reported in patients with radiation-induced bladder symptoms and have shown associations with symptom severity in small exploratory or pilot cohorts [56]. Several studies of pelvic irradiation cohorts and RC/IC-type bladder disorders also show altered urinary inflammatory signatures relative to controls [57]. In RC, persistent low-grade inflammation is believed to arise from continued DAMP signaling, macrophage recruitment, and radiation-induced senescent urothelial cells that secrete pro-inflammatory SASP mediators [41], a which has been reported in association with increased urinary cytokine levels in increased urinary cytokine levels. Although cytokine assays (e.g., ELISA, multiplex immunoassays) are straightforward, they are prone to pre-analytical variability (e.g., timing of collection, dilution, diuresis, storage). Because inflammatory cytokines are broadly elevated across many bladder conditions (e.g., infection, interstitial cystitis, tumor-associated inflammation), they cannot independently distinguish RC from infection, malignancy, or interstitial cystitis without multimodal or longitudinal clinical correlation. Larger, prospective studies (e.g., the RABBIO protocol) are underway to correlate cytokine kinetics with radiation therapy (RT) dosing and symptoms [13]. Moreover, radiation-induced cytosolic DNA activates the cGAS–STING pathway, amplifying type I interferons and proinflammatory cytokines such as IL-6, IL-8, TNFα, and CXCL10 that can be detected in urine [58].

### 3.2. Oxidative Stress and DNA-Damage Markers

Ionizing radiation generates reactive oxygen species (ROS) through multiple mechanisms, beginning with the immediate radiolysis of water [36] and continuing through delayed mitochondrial dysfunction, which in turn promotes electron leakage from the respiratory chain and sustained superoxide generation [59]. In parallel, radiation activates NADPH oxidases, particularly NOX2 and NOX4, via inflammatory cytokines and TGF-β–dependent pathways, further amplifying ROS production [60]. Together, these mitochondrial- and NOX-derived ROS contribute to cumulative oxidative injury, driving chronic urothelial dysfunction and late fibrotic remodeling, processes reflected by increased urinary oxidative stress markers [61].

Widely studied urinary oxidative biomarkers include 8-hydroxy-2′-deoxyguanosine (8-OHdG), a marker of oxidative DNA damage, and malondialdehyde (MDA), a lipid peroxidation product. Elevated urinary 8-OHdG has been described in chronic bladder disorders and in pilot RC-related work, and increases in urinary MDA/other oxidative metabolites have been reported in bladder ischemia and related models. These markers have been reported in association with symptom burden or histologic injury in small exploratory studies [62]. Chronic radiation exposure can maintain oxidative stress through PARP1 activation and mitochondrial insufficiency, mechanisms that reinforce tissue hypoxia and promote fibroblast activation [63]. A few methods could be used to analyze these markers, including ELISA and mass spectrometry; inter-study differences arise from assay selection and urine normalization (creatinine vs. specific gravity). Oxidative markers are sensitive but remain at a primarily exploratory and associative stage, with evidence derived from preclinical radiation-injury models or limited human pilot cohorts; thus, their clinical interpretation is constrained by poor specificity when used alone, as similar elevations occur in infection, ischemia, aging, or malignancy-associated oxidative injury [62].

### 3.3. Endothelial and Vascular-Injury Markers

Radiation causes endothelial cell damage, capillary rarefaction, and microvascular remodeling processes central to hemorrhagic and ischemic phenotypes of late RC. Markers of endothelial activation and angiogenesis released into urine/serum may therefore signal vascular involvement [41]. Vascular injury is a hallmark of late RC and has been associated with changes in urinary and serum markers of endothelial activation. Radiation induces endothelial apoptosis via the acid sphingomyelinase–ceramide pathway, leading to capillary rarefaction, impaired perfusion, and tissue hypoxia [64]. Candidate urinary (and paired serum) markers include VEGF, von Willebrand factor (vWF), plasminogen activator inhibitor-1 (PAI-1) and molecules tied to coagulation and matrix remodeling. Some retrospective analyses have reported associations between elevated PAI-1 and matrix-remodeling proteins with later hemorrhagic outcomes, and small studies have observed urine VEGF/vWF alterations in patients with severe RT bladder toxicity. However, evidence remains preliminary and often mixed [65].

Vascular markers can be informative for hemorrhagic or ischemic risk stratification but may be confounded by systemic vascular disease, anticoagulation, or concurrent pelvic pathology. Paired serum-urine sampling and longitudinal profiling improve interpretability [66].

### 3.4. Extracellular Vesicles (EVs) and microRNAs

Extracellular vesicles including exosomes and microvesicles are membrane-bound particles released by epithelial, endothelial, immune, and stromal cells that carry proteins, lipids, and nucleic acids reflective of their cell of origin. Because EV cargo is actively packaged and relatively stable in urine, EV-based profiling offers potential advantages over freely soluble urinary biomarkers, including enhanced mechanistic specificity and improved temporal stability, particularly during phases of subclinical disease activity. Small RNAs (siRNAs, miRNAs) within EVs regulate fibrosis, inflammation and cell death pathways implicated in RC [67].

Urinary EVs arise from multiple anatomical sources, including renal tubular and glomerular cells, prostate epithelium, immune cells, and critically for radiation cystitis bladder urothelial and endothelial cells [68]. This biological heterogeneity represents both an opportunity and a challenge. While bladder-derived EVs may directly reflect radiation-induced urothelial injury, vascular damage, or fibrotic remodeling, EV cargo can also be influenced by upstream renal pathology, systemic inflammation, aging, metabolic disease, malignancy, and treatment-related factors [41,67,69]. Therefore, careful clinical phenotyping and, where feasible, enrichment for bladder-relevant EV subpopulations are essential for meaningful interpretation in RC.

Ionizing radiation alters EV biogenesis and cargo composition through several well-characterized mechanisms. DNA damage and p53 activation promote EV release as a means of cellular stress signaling and disposal of damaged molecular material [70]. Hypoxia induces HIF-1α–regulated EV microRNAs, such as miR-210, which are linked to ischemic and angiogenic responses [71]. In parallel, activation of TGF-β and Wnt signaling pathways modifies EV-associated profibrotic microRNAs, including miR-21 and members of the miR-200 family, which regulate epithelial–mesenchymal transition and fibroblast activation [72]. Neutrophil activation further contributes EVs enriched in inflammatory and myeloperoxidase-associated proteins (MPO), reflecting innate immune involvement in radiation-induced bladder injury [73]. Radiation enhances EV biogenesis via ESCRT and ceramide-dependent pathways, enriching urine with EV-associated proteins and miRNAs reflective of urothelial stress [74]. Collectively, these mechanisms enrich urine with EV-associated cargo that mirrors urothelial stress, vascular injury, and evolving fibrotic remodeling.

Current evidence supporting EV-based biomarkers in RC is largely associative and derived from pilot clinical cohorts and translational studies, rather than from validated clinical assays [75]. Several reports have demonstrated increased urinary EV concentrations following pelvic radiotherapy and associative links between EV proteomic or miRNA signatures and subsequent bladder toxicity severity [76,77,78]. Proteomic analyses of urinary EVs have implicated neutrophil-derived proteins and immune-related pathways in bladder radiotoxicity, while longitudinal studies suggest that pre- or early post-radiotherapy EV profiles may be associated with an increased risk of late bladder toxicity [69]. However, these findings require replication in larger, multicenter cohorts before clinical implementation.

Methodological variability remains a major barrier to translation. EV isolation techniques, including ultracentrifugation, size-exclusion chromatography, precipitation-based kits, and microfluidic platforms, yield EV populations with differing purity and size distributions [79]. Similarly, normalization strategies (urine volume, creatinine, EV particle count, or total protein) are not standardized and can substantially affect reported associations [80]. Adherence to community reporting standards such as the Minimal information for studies of extracellular vesicles (MISEV) guidelines is therefore essential to improve reproducibility and cross-study comparability.

Compared with soluble urinary cytokines or oxidative stress markers, EVs may offer enhanced tissue specificity and temporal stability, particularly during the latent phase of RC when overt inflammation is minimal but molecular injury continues to evolve [81]. This positions EVs as a potentially valuable modality for detecting subclinical disease progression and for distinguishing acute inflammatory toxicity from delayed fibrotic re-modeling. However, they are biologically heterogeneous; renal disease, aging, malignancy, systemic inflammation, metabolic disease, and pelvic comorbidities can alter EV-miRNA or EV-protein composition and confound attribution to bladder radiation injury. Ongoing clinical and translational studies incorporating paired serum-urine EV profiling and longitudinal sampling are beginning to define how EV-based biomarkers could be integrated into risk stratification and monitoring frameworks for RC. If validated, EV signatures may ultimately support biomarker-guided surveillance strategies or early intervention trials targeting fibrosis, vascular injury, or chronic inflammation. Several ongoing clinical and translational studies are investigating urinary extracellular vesicle profiles, including paired serum–urine analyses, to assess their association with radiation-induced bladder toxicity and long-term cystitis outcomes. However, these studies represent ongoing clinical and translational efforts to evaluate urinary extracellular vesicles as biomarkers of radiation-induced bladder toxicity and progression (Table 1).

### 3.5. Proteomic and Metabolomic Biomarkers

Radiation-mediated tissue injury affects metabolism, extracellular matrix composition, immune cascades and cell death pathways that can be captured by unbiased proteomic and metabolomic profiling of urine. These high-throughput approaches can discover multi-marker panels that reflect the multifactorial pathophysiology of RC [14]. Loss of tight junction integrity (claudins, ZO-1, occludin) facilitates passive release of intracellular proteins and extracellular matrix fragments into urine [82]. Urinary proteomics studies have identified differentially expressed proteins involved in ECM remodeling, complement activation and immune signaling in patients post-RT. Metabolomics, like capillary electrophoresis-MS, has highlighted perturbations in amino-acid metabolism and small molecules (examples reported include betaine, N,N-dimethylglycine and others) that distinguished patients with early RC symptoms in a prostate radiotherapy cohort. These omics studies often yield candidate multi-marker panels with associative discriminatory potential rather than validated clinical classifiers [14]. Although it has the capability of broad discovery, it needs rigorous normalization, large validation cohorts, and bioinformatic integration. Multi-omics integration and machine learning approaches are promising for building predictive classifiers but require multicenter datasets to avoid overfitting [75].

### 3.6. Hematuria-Related and Urothelial Cell Markers

Hematuria (micro or gross) is a frequent and clinically important manifestation of RC, especially late hemorrhagic cystitis. Urinary detection of blood-derived proteins or urothelial cell debris can therefore be used to grade bleeding severity or identify active mucosal disruption [41]. Urinary hemoglobin/hematin, urobilinogen derivatives, fibrin degradation products, and cytologic detection of urothelial cells or atypia are the commonly measured signals. While these markers document bleeding and mucosal sloughing, urinary hematuria-related proteins overlap with multiple bladder conditions (infection, tumor recurrence, calculi, or intrinsic permeability disorders), and therefore primarily indicate active mucosal disruption or bleeding, rather than radiation etiology in isolation. thus, they are most useful when interpreted in clinical context and combined with more mechanistic biomarkers [14]. Hematuria quantification is simple and clinically actionable, but by itself it does not illuminate underlying mechanistic drivers (inflammation vs. ischemia vs. tumor) or predict progression. Combining hematuria metrics with molecular markers such as EV proteins and endothelial markers improves diagnostic specificity [66].

Table 1 summarizes potential urinary biomarkers associated with radiation cystitis (RC), organized by biological process, biomarker type, molecular function, analytical methods, clinical phase relevance, and methodological workflows. It integrates mechanistic insights with practical considerations for biomarker acquisition, processing, and interpretation, highlighting translational potential across acute, transitional, and chronic phases of RC.

**Table 1:** Comparative overview of urinary biomarker classes in radiation cystitis. The table summarizes major urinary biomarker categories, their biological relevance, analytical methods, disease-phase association, and key limitations.

Biological Process	Biomarker Type	Key Examples	Function	Analytical Methods	Clinical Phase	Specificity & Limitations	Key Studies
Inflammation	Cytokines & Chemokines	IL-6, IL-8, TNF-α, MCP-1	Mediate acute inflammation	ELISA, multiplex bead assays	Acute	Non-specific; timing and dilution confound	[13,57]
Oxidative Stress	Oxidative DNA & lipid damage	8-OHdG, MDA	ROS-damage markers; cumulative injury signal	ELISA, LC-MS/MS, spectrophotometry	Acute → Chronic	Non-specific; infection/ischemia/sampling confound	[61,62]
Vascular Injury	Endothelial & angiogenic markers	VEGF, vWF, PAI-1, TIMP-1/2	Endothelial activation and remodeling	ELISA, immunoblotting; paired serum-urine	Chronic	Confounded by systemic vascular disease and comorbidities	[12,66,69]
Fibrosis & Remodeling	ECM & fibrogenic proteins	TGF-β, CTGF, TIMPs, PAI-1	Fibroblast activation and ECM deposition	ELISA, proteomics, immunoassays	Chronic	Overlap with fibrotic disorders; longitudinal validation needed	[8,14]
Cellular Damage	Hematuria-related proteins	Hemoglobin, urothelial debris	Reflect mucosal disruption and bleeding	Dipstick, cytology, spectrometry	Acute → Chronic	Non-specific; interpret in clinical context	[67]
EVs & miRNAs	EVs cargo	miR-21, miR-200 family, neutrophil proteins	Carrying regulatory RNAs and proteins from bladder	Ultracentrifugation, NTA, qPCR, proteomics	latent → Chronic	Technically demanding; lack of standardization across studies	[67,83]
Metabolic Shifts	Small molecules & metabolites	Betaine, tartrate, homocarnosine	Reflect metabolic perturbations and oxidative stress	CE-MS, LC-MS, metabolomics platforms	Acute → latent	Normalization critical; usually panel-based.	[14,84]

Biomarker categories include inflammatory cytokines, oxidative stress markers, endothelial and angiogenic proteins, fibrogenic mediators, hematuria-related proteins, extracellular vesicle (EV) cargo, and small-molecule metabolites. Analytical methods span immunoassays, mass spectrometry, cytology, and omics platforms. Methodological workflows reflect current best practices in urine collection, normalization, and assay implementation, with emphasis on reproducibility and clinical applicability. Key studies cited represent foundational and emerging evidence supporting biomarker relevance in RC pathophysiology and risk stratification.

## 4. Future Directions

### 4.1. Standardization, Study Design, and Biobanking

Translating urinary biomarkers from discovery to clinical practice requires strict standardization throughout the entire pipeline. This includes harmonizing urine collection methods (e.g., timing of collection, midstream versus catheterized samples), standardizing pre-analytical handling (such as the use of additives, centrifugation, storage conditions, and duration), applying consistent normalization methods (e.g., creatinine or specific gravity), and ensuring reliable analytical platforms (assay selection and calibration). Multi-center prospective cohorts with pre-treatment baseline sampling and longitudinal follow-up are essential to capture inter-individual variability and time-dependent biomarker kinetics after radiotherapy. The RABBIO prospective protocol exemplifies this approach by pairing serial urine and blood sampling with digital patient-reported outcomes during pelvic radiotherapy [75]. Key steps include centralized biobanking with SOPs, standardized clinical endpoints, and collection of covariates that may confound biomarker signals. Multi-center collaborations and data sharing will be critical to achieve sufficient sample sizes for validation and stratified analyses [13]. Urinary biomarker profiles in RC are constrained by substantial inter-patient heterogeneity, including variability driven by radiation dose, fractionation, bladder volume exposure, baseline urothelial health, age, sex, renal and cardiovascular comorbidities, smoking status, hydration dynamics, and concurrent medications (e.g., anticoagulants or anti-inflammatory agents). This heterogeneity limits the generalizability of single-marker findings, reinforcing the need for baseline-normalized, longitudinal, and multi-analyte panel approaches validated across independent, multi-center cohorts [14,48,85].

Extracellular vesicles (EVs) and small RNAs are among the most promising urine-based biomarker modalities due to improved tissue specificity and cargo stability. However, EV isolation and reporting are highly variable across studies; adherence to community standards such as the MISEV guidelines (MISEV2018/MISEV updates) should be mandated in discovery and validation studies to ensure reproducibility (clear reporting of separation methods, particle sizing, protein markers, and negative controls). Standardized EV workflows will also facilitate cross-study meta-analyses and clinical translation [83].

### 4.2. Multi-Omics Integration and Machine Learning

Single biomarkers will likely be insufficient for the heterogeneous biology of RC. Integrative multi-omics approaches combining cytokine panels, proteomics, metabolomics, EV proteomics/miRNA, and conventional clinical metrics are more likely to capture the multifactorial processes including inflammation, oxidative stress, vascular injury and fibrosis, that underlie different clinical phenotypes [41]. Machine learning models trained on well-curated, multi-center datasets can identify stable multi-marker signatures and generate risk scores for early toxicity, hemorrhagic risk, or progression to contracted bladder. Crucially, models should be developed with pre-specified validation sets, rigorous cross-validation, and external cohort validation to avoid overfitting [84].

### 4.3. Paired Sampling and Longitudinal Kinetics

Paired serum-urine sampling and repeated longitudinal urine sampling (baseline, during RT, early post-RT, and long-term follow-up) are critical to understand whether urinary signals reflect local bladder pathology or systemic responses [86,87,88,89]. Longitudinal trajectories can help distinguish acute transient inflammation from persistent changes that predict late fibrosis or hemorrhage. Studies such as RABBIO that couple objective biomarker data with digital symptom monitoring are models for this design [75]. However, because RC shares molecular injury outputs with infection, malignancy, ischemic bladder disorders, and intrinsic permeability syndromes, the most interpretable biomarkers are those showing radiation-aligned temporal kinetics or direct mechanistic linkage to RT-activated pathways when validated across independent, multicenter cohorts.

### 4.4. Prioritizing Mechanistic Biomarkers That Are Therapeutically Actionable

Biomarkers that reflect targetable pathways, such as TGF-β-driven fibrosis, VEGF-mediated neovascular fragility, and persistent oxidative stress, should be prioritized because they can inform biomarker-guided interventions or repurposing of existing agents including antifibrotics, anti-angiogenic modulators, antioxidants, or targeted intravesical therapies [90,91,92,93,94,95,96]. For example, urinary elevations of fibrotic mediators (PAI-1, TIMP-1/2) and angiogenic factors (VEGF-A) observed in prostate cancer survivors with symptomatic RC suggest axes that could be trialed as both biomarkers and therapeutic targets. Validation of such markers will enable early-stage biomarker-directed intervention trials [12].

For urinary biomarkers to reach clinical practice, they must show analytic and clinical validity linked to meaningful outcomes. Early engagement with regulators and payers, clear clinical use-cases, and cost-effectiveness analyses will accelerate translation. Prospective trials that randomize biomarker-positive patients to preventive or escalated strategies represent a critical next step [97,98,99].

The translational pathway for urinary biomarkers in radiation cystitis should proceed linearly from (i) mechanistic biomarker discovery in preclinical or exploratory human cohorts, to (ii) analytical validation and standardized assay harmonization, to (iii) multicenter clinical validation in large, baseline-normalized longitudinal cohorts, followed by (iv) integration into biomarker-guided interventional or preventive clinical trials, regulatory qualification, and eventual payer adoption for clinical implementation.

## Data Availability

No new data were created or analyzed in this study. Data sharing is not applicable to this article.
